# Interactions Between Natural Herbicides and Lipid Bilayers Mimicking the Plant Plasma Membrane

**DOI:** 10.3389/fpls.2019.00329

**Published:** 2019-03-18

**Authors:** Simon Lebecque, Laurence Lins, Franck E. Dayan, Marie-Laure Fauconnier, Magali Deleu

**Affiliations:** ^1^TERRA, Laboratory of Molecular Biophysics at Interfaces, Gembloux Agro-Bio Tech, University of Liège, Gembloux, Belgium; ^2^TERRA – AgricultureIsLife, Gembloux Agro-Bio Tech, University of Liège, Gembloux, Belgium; ^3^Department of Bioagricultural Sciences and Pest Management, Colorado State University, Fort Collins, CO, United States; ^4^General and Organic Chemistry Laboratory, Gembloux Agro-Bio Tech, University of Liège, Gembloux, Belgium

**Keywords:** plasma membrane, lipid, natural herbicides, phytotoxicity, allelopathy, sorgoleone, sarmentine, nonanoic acid

## Abstract

Natural phytotoxic compounds could become an alternative to traditional herbicides in the framework of sustainable agriculture. Nonanoic acid, sarmentine and sorgoleone are such molecules extracted from plants and able to inhibit the growth of various plant species. However, their mode of action is not fully understood and despite clues indicating that they could affect the plant plasma membrane, molecular details of such phenomenon are lacking. In this paper, we investigate the interactions between those natural herbicides and artificial bilayers mimicking the plant plasma membrane. First, their ability to affect lipid order and fluidity is evaluated by means of fluorescence measurements. It appears that sorgoleone has a clear ordering effect on lipid bilayers, while nonanoic acid and sarmentine induce no or little change to these parameters. Then, a thermodynamic characterization of interactions of each compound with lipid vesicles is obtained with isothermal titration calorimetry, and their respective affinity for bilayers is found to be ranked as follows: sorgoleone > sarmentine > nonanoic acid. Finally, molecular dynamics simulations give molecular details about the location of each compound within a lipid bilayer and confirm the rigidifying effect of sorgoleone. Data also suggest that mismatch in alkyl chain length between nonanoic acid or sarmentine and lipid hydrophobic tails could be responsible for bilayer destabilization. Results are discussed regarding their implications for the phytotoxicity of these compounds.

## Introduction

Weeds management is a key point for an efficient agriculture and nowadays it mainly relies on the use of herbicides. Unfortunately, traditional herbicides are becoming ineffective because of the evolution of resistance, and there are some public concern related to the potential environmental and health impact of pesticides. In this context, identification of natural phytotoxic compounds with new modes of action is a promising way to reach a more sustainable agriculture. Much effort has been made in this direction, which enabled isolation and identification of numerous molecules with an herbicidal activity (Dayan and Duke, 2014). However, a comprehensive understanding of the mechanisms underlying the phytotoxicity of such molecules is mandatory prior to their safe use on the field. In this paper, three naturally occurring compounds, namely nonanoic acid, sarmentine and sorgoleone were considered.

**FIGURE 1:**
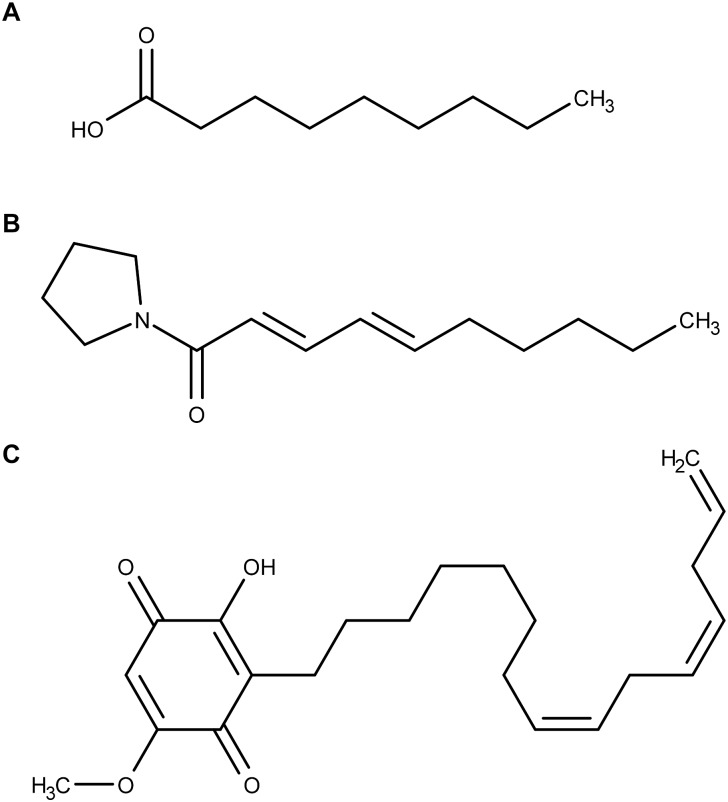
Molecular structures of **(A)** nonanoic acid, **(B)** sarmentine, and **(C)** sorgoleone.

Nonanoic acid (Figure 1A) (also known as pelargonic acid) is produced by geranium (*Pelargonium* spp.). It has been used for years as a contact herbicide quickly leading to necrotic lesions on aerial parts of the target plants. A comparison of the phytotoxicity of various fatty acids as a function of their carbon chain length demonstrated that fatty acids with C9 to C11 chain lengths where more active than fatty acids with shorter or longer carbon chains (Fukuda et al., 2004). According to Lederer et al. (2004), their phytotoxicity is due to fatty acids’ ability to penetrate and destabilize plasma membranes. However, there is no study accurately investigating the interactions between nonanoic acid and biological membranes.

Sarmentine (Figure 1B), a recently isolated amide from long pepper (*Piper longum* L.) fruit, has herbicidal activity on a variety of plant species (Huang et al., 2010). Plants treated with sarmentine develop very similar symptoms to those observed on plants treated with decanoic acid. The authors suggested that sarmentine phytotoxicity is primarily exerted through membrane destabilization related to its alkyl chain length. More recent work demonstrated that sarmentine is likely to have multiple mechanisms of action, including inhibition of photosystem II and inhibition of enoyl-ACP reductase (Dayan et al., 2015). At the same time, it was proven that sarmentine induces destabilization of plasma membrane from cotyledon disks assays (Dayan et al., 2015). This effect was also observed with nonanoic acid, again suggesting that both molecules share at least one mode of action. Sarmentine was, however, much more potent in inducing destabilization of membrane integrity (Dayan et al., 2015).

Sorgoleone (Figure 1C) is an allelochemical compound exuded from *Sorghum bicolor* (L.) Moench roots (Einhellig and Souza, 1992; Dayan et al., 2010). This molecule inhibits photosynthesis by interrupting the photosynthetic electron transport in isolated chloroplasts (Nimbal et al., 1996; Gonzalez et al., 1997). However, sorgoleone lipophilicity prevents its translocation from roots to foliage and thus it is not able to reach its putative site of action (Hejl and Koster, 2004; Dayan et al., 2009). Moreover, even when it is directly applied on the foliage, sorgoleone is ineffective on photosynthesis of mature plants (Dayan et al., 2009). *In planta* inhibition of photosynthesis seems to only occur in young seedlings. Hence, it is supposed that sorgoleone exhibits its toxicity toward older plants through other modes of action. For instance, Hejl and Koster observed that root H^+^-ATPase is inhibited by sorgoleone (Hejl and Koster, 2004). They suggested that the resulting solute and water uptake alteration could contribute to the phytotoxicity of the herbicidal molecule.

Nonanoic acid, sarmentine and sorgoleone have a broad spectrum toxicity (Einhellig and Souza, 1992; Dayan et al., 2012, 2015) and share structural similarities: their molecular weight is low (<400 g/mol) and they all possess highly hydrophobic alkyl tails and somewhat polar heads. Given these common features, we can make the assumption that they could interact with lipid membranes and that their efficacy might be linked to this property. Concerning nonanoic acid and sarmentine, such interactions with cellular membranes are thought to be at least partly responsible for their phytotoxicity, but the molecular understanding and details are lacking. The putative interactions occurring between sorgoleone and lipid bilayers is also evident since its target site in photosystem II is embedded in the thylakoid membrane of chloroplasts. Due to its chemical structure and its low mobility within plant tissues supposedly due to a high lipophilicity, investigating the behavior of sorgoleone in the presence of lipid membranes could bring valuable information about its mechanism of action.

Biological membranes are very complex structures composed of numerous lipid species forming a bilayer in which a variety of proteins are embedded. Because of this complexity, model membranes are generally used to mimick biological membranes. Such model membranes are made of one or a few representative lipid species forming structures such as monolayers, bilayers or vesicles that share common features with biological membranes (Deleu et al., 2014). In this work, interactions between each of the above herbicidal molecules and plant model membranes are explored. At first, the effect of the herbicidal compounds on the lipid bilayer order and fluidity is studied at various temperatures with fluorescent measurements. Then, a thermodynamic characterization of the interactions occurring between the chemicals under investigation and lipid vesicles is carried out by using ITC. Finally, molecular mechanisms of these interactions are explored by performing MD simulations.

## Materials and Methods

### Chemicals

1,2-Dipalmitoyl-sn-glycero-3-phosphocholine (DPPC) and PLPC were purchased from Avanti Polar Lipids, Inc. Sarmentine was purchased from Toronto Research Chemicals Inc. All other chemicals, including nonanoic acid, laurdan and DPH were purchased from Sigma-Aldrich. Sorgoleone was extracted from *Sorghum bicolor* (L.) Moench as described previously (Dayan et al., 2009).

### Liposome Preparations

For laurdan generalized polarization experiments, MLVs prepared as follows were used. A small volume of DPPC dissolved into chloroform-methanol (2:1) was either put alone or mixed with small amounts of nonanoic acid, sarmentine or sorgoleone (dissolved into 2:1 chloroform-methanol) into a round bottom tube to reach a lipid:herbicide molar ratio of 5:1. Solvent was then evaporated under a gentle stream of nitrogen. The tube was kept overnight under vacuum to remove solvent traces. The resulting film was then hydrated with 10 mM TRIS - HCl buffer at pH 7 prepared from Milli-Q water. The tube was maintained at a temperature (∼51°C) well above the transition phase temperature of the lipid for at least 1 h and vortexed for 1–2 min every 10 min. Thereafter, the MLV suspension underwent 5 freeze-thaw cycles.

For DPH anisotropy measurements, pure DPPC LUVs were prepared by following the same procedure followed by 11 passages through polycarbonate filters with a pore diameter of 100 nm at a temperature (∼51°C) well above the transition phase temperature of the lipid.

For ITC, LUVs were used. Small amounts of lipids (PLPC) were dissolved into chloroform-methanol (2:1) in a round-bottom flask. A rotary evaporator was used to remove solvent under low pressure and the flask was then kept overnight under vacuum to remove solvent traces. The lipid film was then hydrated with 10 mM TRIS - HCl buffer at pH 7 prepared from Milli-Q water. The flask was maintained at a temperature (∼37°C) well above the transition phase temperature of the lipid for at least 1 h and vortexed for 1–2 min every 10 min. Thereafter, the MLV suspension underwent 5 freeze-thaw cycles. In order to get LUVs, MLV suspension was then extruded 11 times through polycarbonate filters with a pore diameter of 100 nm.

### Laurdan Generalized Polarization

Fluorescence of laurdan in DPPC (either pure or mixed with one of the studied molecules in a lipid:herbicide molar ratio of 5:1) MLVs was monitored at various temperatures with a Perkin Elmer LS50B fluorescence spectrometer. Lipid concentration was 50 μM and 1 μL of a stock solution of laurdan in DMSO was added to 3 ml of MLV suspension to reach a lipid:probe molar ratio of 200:1. A corresponding blank was prepared by adding 1 μL of pure DMSO instead of the laurdan stock solution to 3 ml of MLV suspension from the same batch. Both preparations were kept at a temperature (∼51°C) well above the phase transition temperature of DPPC for 1 h in the dark to let the probe partition into the MLVs. Samples were then placed in 10 mm path length quartz cuvettes under continuous stirring while the cuvette holder was thermostated with a circulating bath. Samples were equilibrated at the initial temperature of 31°C for 15 min before the first measurements were performed. For the measurements, excitation wavelength was set to 360 nm, and at least 12 measurements of emission intensities at 440 and 490 nm were recorded and averaged for each sample at each temperature. An emission spectrum from 400 to 600 nm was also recorded for each sample-temperature combination. Excitation slit width was set to 2.5 nm, while emission slit width ranged from 4 to 6.5 nm. When all measurements at a given temperature were recorded, temperature was increased by 4, 3, 2, or 1 degree(s) and samples were equilibrated for 10 min once the new temperature was reached. Generalized polarization (GP) of laurdan was then calculated according to (Parasassi et al., 1991; Jay and Hamilton, 2017):

(1) $$\text{GP}=\frac{I_{440}-I_{490}}{I_{440}+I_{490}}$$

where *I*_440_ and *I*_490_ are the blank-subtracted emission intensities at 440 and 490 nm, respectively. All experiments were carried out at least three times, each time with liposomes prepared fresh daily.

### DPH Anisotropy

Fluorescence anisotropy measurements of DPH in DPPC LUVs either in the gel phase (at 35°C) or in the liquid phase (at 50°C) were performed with a Perkin Elmer LS50B fluorescence spectrometer equipped with polarizers. To prepare the samples, stock solutions of nonanoic acid, sarmentine or sorgoleone dissolved into DMSO at various concentrations were prepared. Four μL of each of these solutions were added to aliquots (3 mL) of 50 μM DPPC LUV suspension to reach final concentrations of 10, 25, 50, and 100 μM of natural herbicide. For pure DPPC sample, 4 μL of DMSO were added to an aliquot of 50 μL DPPC LUV suspension. One μL of a stock solution of DPH dissolved into DMSO was then added to each sample to reach a lipid:probe molar ratio of 200:1. Corresponding blanks were prepared by adding 1 μL of pure DMSO instead of the DPH stock solution. Each preparation was kept at a temperature (∼51°C) well above the phase transition temperature of DPPC for 1 h in the dark to let the probe and the molecules partition into the LUVs. Samples were then placed in 10 mm path length quartz cuvettes under continuous stirring while the cuvette holder was thermostated with a circulating bath. Samples were equilibrated at the desired temperature for 15 min before the measurements were performed. For the measurements, excitation and emission wavelengths were set to 360 and 426 nm, respectively. Excitation slit width was set to 2.5 nm, while emission slit width ranged from 4.5 to 12 nm. Both excitation and emission polarizers can be either in a vertical (V) or a horizontal (H) orientation. For each sample, at least 12 measurements were recorded and averaged for each of the four possible combinations of polarizers orientations. The anisotropy parameter, *r*, was then calculated according to previous papers (Harris et al., 2002; Lakowicz, 2006):

(2) $$r=\frac{I_{\mathrm{VV}}-G\times I_{\mathrm{VH}}}{I_{\mathrm{VV}}+2\times G\times I_{\mathrm{VH}}}$$

where *I_VV_* and *I_VH_* are the blank-subtracted emission intensities with the excitation and the emission polarizers oriented as indicated by the subscripts in this order (for instance, *I_VH_* corresponds to the blank-subtracted emission intensity with the excitation polarizer in a vertical orientation and the emission polarizer in a horizontal orientation) and *G* is a correction factor obtained as follows:

(3) $$G=\frac{I_{\mathrm{HV}}}{I_{\mathrm{HH}}}$$

where *I_HV_* and *I_HH_* are the blank-subtracted emission intensities with the excitation and the emission polarizers oriented as indicated by the subscripts in this order. All experiments were carried out at least three times. Measurements at 50°C were carried out with liposomes prepared fresh and measurements at 35°C were performed within 3 days following liposomes preparation.

### Isothermal Titration Calorimetry

The ITC measurements were performed with a VP-ITC from Microcal (Microcal Inc., Northampton, MA, United States). The sample cell contained 1.4565 mL of a solution of nonanoic acid (500 μM), sarmentine (75 μM) or sorgoleone (75 μM) dissolved in the same buffer as the LUV suspension. Reference cell was filled with mQ water. Small aliquots of LUV suspension were added to the sample cell with a software-controlled syringe. The first injection was 2 μL and was not taken into account for data treatment. It was followed by 28 successive additions of 10 μL, with an interval of 600 s. For nonanoic acid and sarmentine, a lipid concentration of 5 mM was used. As sorgoleone affinity for lipid vesicles was higher than the other compounds tested, lipid concentration was lowered to 1 mM for ITC experiments involving this molecule. For data treatment, Origin 7.0 software was used as previously described (Heerklotz and Seelig, 2000; Zakanda et al., 2012) in order to fit the cumulative heats of binding to the equation:

(4) $$\sum_{k=1}^{i}\delta h_{k}=\Delta H_{D}^{W\to b}V_{\mathrm{cell}}C_{A}^{0}\frac{{\mathrm{KC}}_{L}^{0}}{1+{\mathrm{KC}}_{L}^{0}}$$

where δ*h_k_* is the heat produced after each injection, Δ*H_D_*^*w*→*b*^ is the difference in molar enthalpy originating from the transfer of the herbicidal molecules from the aqueous phase to the bilayer membrane, *V_cell_* is the volume of the sample cell, *C*^0^_*A*_ and *C*^0^_*L*_ are the total herbicide and lipid concentration, respectively, in the cell after *i* injections and *K* is the partition constant. Temperature was maintained at 26°C during the course of the experiments. Data analysis was done using Origin software (version 7.0) from Microcal.

### Molecular Dynamics

Simulations have been performed with GROMACS 4.6.7 and the united atom GROMOS 53a6 force field (Oostenbrink et al., 2004) with three replicates. Topologies of the natural herbicides have been manually refined from Automatic Topology Builder’s results (Malde et al., 2011). A PLPC topology derived from Berger Lipids force field (Berger et al., 1997) and developed by Peter Tieleman’s group (Bachar et al., 2004) was used. It is available for download on its website (Tieleman, 2018). Bilayers containing 140 PLPC molecules and 28 herbicide (nonanoic acid, sarmentine or sorgoleone) molecules (lipid:herbicide molar ratio of 5:1) were generated and hydrated by using Memgen (Knight and Hub, 2015). Each system was solvated with SPC water (Hermans et al., 1984). Nonanoic acid being unprotonated in our simulations, 28 K^+^ ions (Gurtovenko and Vattulainen, 2008; Cordomí et al., 2009) were added to systems containing nonanoic acid molecules to get an overall charge of zero. All systems firstly underwent a 100 ps NVT equilibration followed by a 1 ns NPT equilibration during which herbicides were under position restraints. 250 ns production runs were then performed from which the first 150 ns were considered as equilibration time and discarded for data analyses. For the production runs, temperature was maintained to an average value of 298K by using the Nose-Hoover thermostat (Nosé, 1984; Hoover, 1985) with a τ_T_ = 0.5 ps. Semi-isotropic pressure (1 bar) was maintained by using the Parrinello-Rahman barostat (Parrinello and Rahman, 1981) with a compressibility of 4.5 × 10^−5^ bar^−1^ and τ_P_ = 2 ps. Electrostatic interactions were treated by using the particle mesh Ewald (PME) method. A cut-off of 1nm was used for Van der Waals interactions. Bond lengths were maintained with the LINCS algorithm (Hess et al., 1997). Trajectories were analyzed with GROMACS tools as well as with homemade scripts and were visually analyzed with VMD (Humphrey et al., 1996) and PYMOL (The PyMOL Molecular Graphics System) software packages.

## Results

### Effect on Bilayer Order and Lipid Phase Transition Temperature

Laurdan is a fluorescent probe that readily partitions at the hydrophilic/hydrophobic interface of bilayers (Bernik et al., 2001; De Vequi-Suplicy et al., 2006). Laurdan fluorescence properties depend on the physical state of the probe environment. When inserted into a lipid bilayer in the gel phase, fluorescence intensity is maximal at an emission wavelength of about 440 nm. As the lipid bilayer undergoes transition to a liquid phase, the maximal fluorescence intensity will occur at higher emission wavelengths. This so-called “red shift” is imputed to an increased presence and mobility of water molecules around the fluorophore (Parasassi et al., 1991; De Vequi-Suplicy et al., 2006) which can be directly linked to the lipid bilayer order (Harris et al., 2002). Laurdan generalized polarization (GP) parameter has been developed in order to conveniently monitor these lipid order changes (Parasassi et al., 1991). Here, laurdan GP is used to study the effect of the natural herbicides on the phase transition temperature (Tm) of DPPC MLVs as well as to investigate their effect on lipid order at the hydrophilic/hydrophobic interface below, around and above the Tm of DPPC.

**FIGURE 2:**
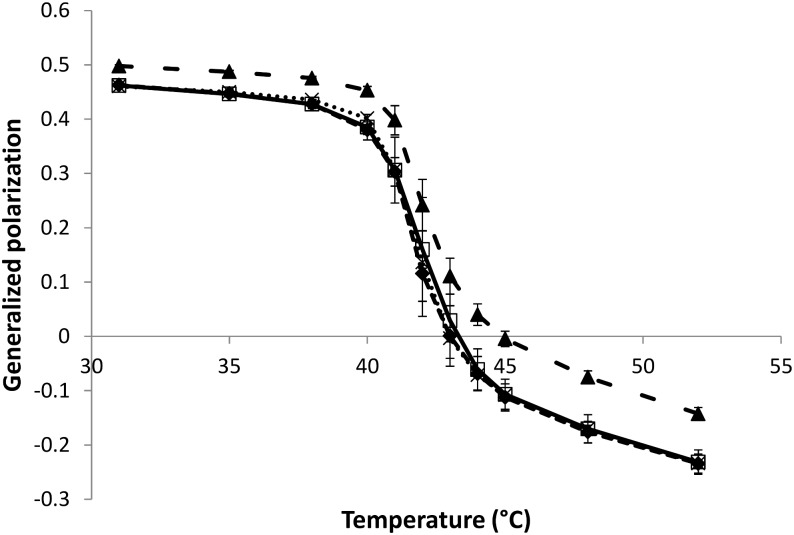
Evolution of laurdan generalized polarization as a function of temperature for DPPC MLVs (50 μM) in the absence or presence (lipid:herbicide molar ratio 5:1) of natural herbicides (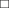: pure DPPC, 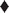: DPPC + nonanoic acid, ×: DPPC + sarmentine, 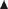: DPPC + sorgoleone).

The temperature dependence of laurdan GP for MLVs made of either pure DPPC or DPPC mixed with a natural herbicide (lipid:herbicide molar ratio of 5:1) is shown in Figure 2. Nonanoic acid and sarmentine do not affect the phase transition temperature of DPPC MLVs at this concentration. On the other hand, DPPC MLVs mixed with sorgoleone exhibit a slightly increased Tm when compared to pure DPPC. However, the cooperativity of the phase transition does not seem to be affected by sorgoleone as no broadening of the curve in this region is detected. In addition, we observe that nonanoic acid and sarmentine do not induce any change in lipid order at any temperature as their respective curves superimpose the pure DPPC curve. For sorgoleone and DPPC mixed liposomes, laurdan GP values are higher both in the gel phase and in the liquid phase when compared to the other MLV compositions. This likely indicates an ordering effect of sorgoleone on DPPC bilayer.

### Study of the Fluidity of the Hydrophobic Core of Bilayer in the Presence of the Natural Herbicides

The DPH is a hydrophobic fluorescent probe that spontaneously and almost exclusively inserts into the hydrocarbon chains region of lipid bilayers (Kaiser and London, 1998) and is then widely used to analyze the microviscosity of this region in artificial membranes (Shinitsky and Barenholz, 1978).

As its excited state lifetime is sufficiently long, DPH can change its orientation before fluorescence emission occurs. The ability of the probe to reorient within the hydrophobic core of the lipid bilayer is directly linked to the microviscosity/fluidity of the hydrocarbon chains of the bilayer (Lentz, 1989; Stott et al., 2008). When the exciting light is polarized, rotational movements of the probe induce a change in polarization for the emission light (Lentz, 1993). The less fluid is the bilayer, the less rotational movements of DPH occur and the more polarized is the emission light, which is translated into a high value of anisotropy parameter r (see Eq. 2, section “Materials and Methods”). Hence, this anisotropy parameter *r* has been monitored to investigate the effect of the natural herbicides at different concentrations on the bilayer fluidity.

**FIGURE 3:**
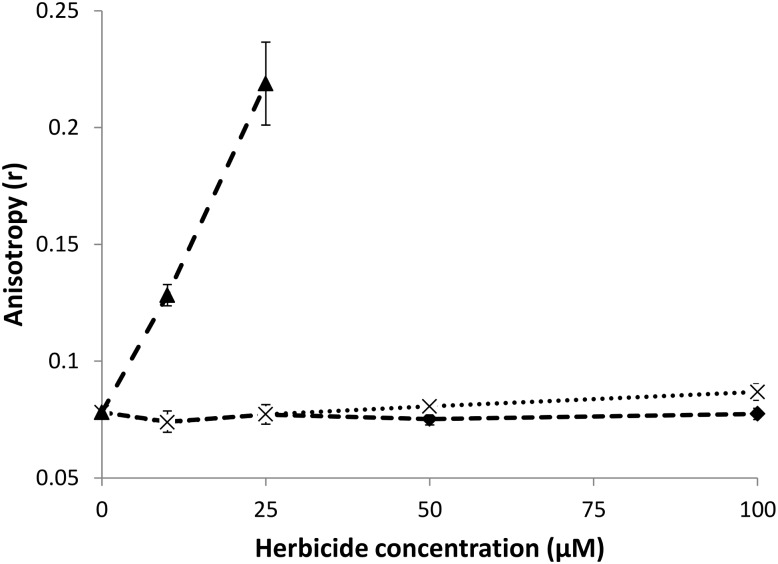
Evolution of DPH anisotropy for DPPC LUVs (50 μM) mixed with increasing concentrations of natural herbicides at 50°C (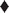: DPPC + nonanoic acid, ×: DPPC + sarmentine, 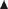: DPPC + sorgoleone).

The evolution of DPH anisotropy for DPPC LUVs (lipid concentration = 50 μM) at 50°C as a function of the concentration of added natural herbicides is shown in Figure 3. At this temperature, DPPC LUVs are in the liquid phase and thus exhibit low *r*-values. Nonanoic acid does not induce any significant change in DPH anisotropy in the range of concentrations tested here (10–100 μM, i.e., a lipid:herbicide molar ratio ranging from 5:1 to 1:2). Sarmentine seems able to slightly increase *r*, but only at the highest concentration used in this study. Sorgoleone, on the other hand, induces a sharp rise of DPH anisotropy even at concentration as low as 10 μM. Albeit less marked, similar trends are observed at 35°C, when liposomes are in the gel phase (see Supplementary Figure S1).

### Partitioning Into Lipid Vesicles

Isothermal titration calorimetry experiments were carried out to study the ability of the natural herbicides to partition into lipid bilayers and to thermodynamically characterize interactions that could occur.

**FIGURE 4:**
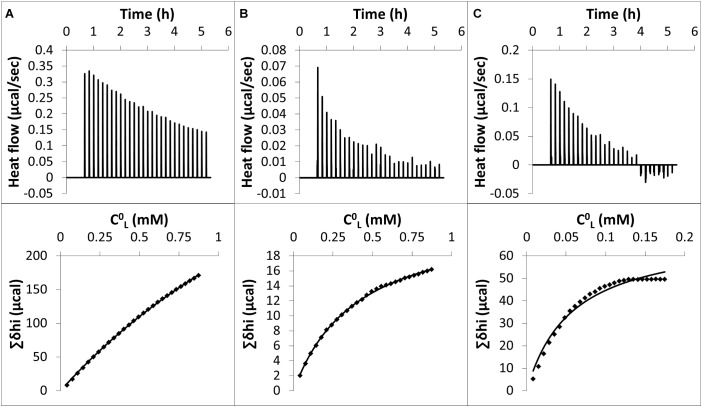
Upper panels: raw data from ITC experiments. Each peak corresponds to a single injection of 10 μL of a PLPC LUV suspension [5 mM for **(A)** and **(B)**, 1 mM for **(C)**] into a solution containing **(A)** 500 μM nonanoic acid, **(B)** 75 μM sarmentine and **(C)** 75 μM sorgoleone at 26°C. LUV suspension and herbicides solutions were buffered at pH 7 with TRIS – HCl. Lower panels: cumulative heats of binding (Σδhi) as a function of the lipid concentration in the cell (*C*^0^_L_). Solid line corresponds to the best fit using the Eq. 4.

Typical raw data of an ITC experiment for nonanoic acid (A), sarmentine (B) and sorgoleone (C) with PLPC LUVs are shown in Figure 4 (upper panels). The corresponding cumulative heats of binding (Σδhi) are also plotted against the lipid concentration in the cell (*C*^0^_L_) (lower panels). Fitting curves are obtained from Eq. 4 and enabled determination of the partition coefficient, *K*, as well as thermodynamic parameters characterizing the interactions, as summarized in Table 1. ITC raw data display a gradual decrease of the positive heat flow signal over the course of the successive injections which is characteristic of a binding event. The thermodynamic parameters (Table 1) indicate that the binding is spontaneous (Δ*G* < 0), endothermic (Δ*H* > 0) and leads to a positive change of entropy (Δ*S* > 0) for all three molecules. The absolute value of entropy change is much higher than the one of enthalpy change, indicating that the binding is entropy-driven. This is probably due to the transfer of the hydrophobic chain of the herbicidal molecules from the water to the hydrophobic core of the liposomes. This is supported by the good correlation observed between the number of carbon atoms in the alkyl chain of each compound and their respective magnitude of TΔS_D_^w→b^. Nonanoic acid has the lowest binding affinity among the studied compounds. Sarmentine partition coefficient is almost one order of magnitude higher than that of nonanoic acid while sorgoleone one is two orders of magnitude larger.

**Table 1:** Thermodynamic parameters characterizing the interactions of herbicidal molecules with PLPC LUVs.

Natural compound	*K* (mM^−1^)	Δ*H*_D_^w→b^ (kJ.mol^−1^)	TΔS_D_^w→b^ (kJ.mol^−1^)	Δ*G*_D_^w→b^ (kJ.mol^−1^)
Nonanoic acid	0.36 ± 0.06	3.32 ± 0.67	27.85 ± 0.6	−24.53 ± 0.48
Sarmentine	1.69 ± 0.57	1.47 ± 0.61	29.77 ± 0.39	−28.3 ± 0.82
Sorgoleone	28.5 ± 8.33	1.79 ± 0.77	37.13 ± 0.68	−35.34 ± 0.8

### Interaction of the Natural Herbicides With the Lipid Bilayer and Their Effect on the Lipid Order Parameter Studied by Molecular Dynamics

**FIGURE 5:**
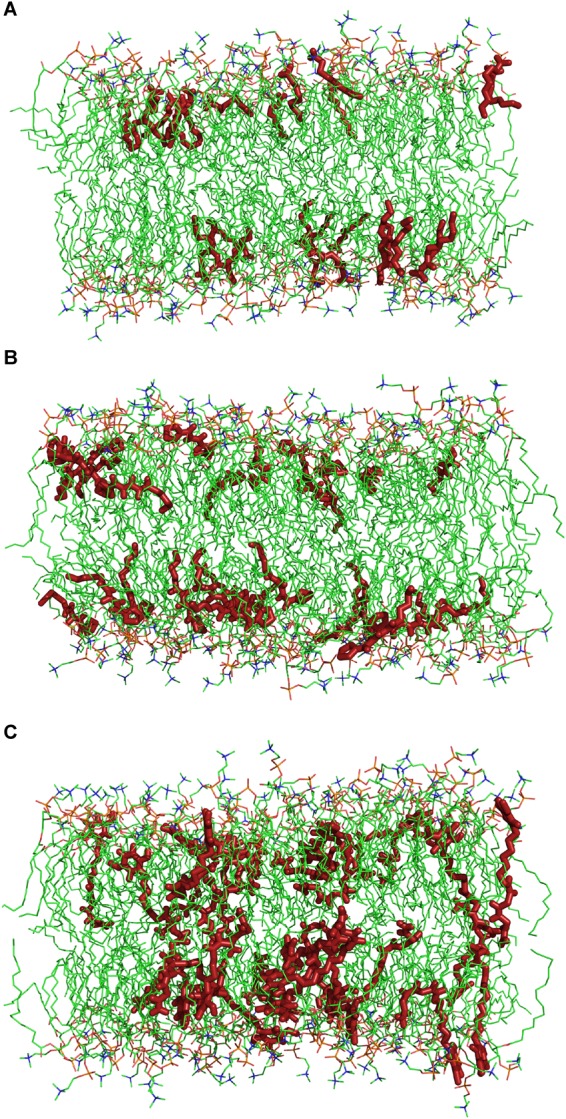
Snapshots after 250 ns simulations of a 140 molecules PLPC bilayer with **(A)** 28 nonanoic acid molecules, **(B)** 28 sarmentine molecules or **(C)** 28 sorgoleone molecules. For sake of clarity, water molecules and ions are omitted. Dark red: herbicidal molecules, green: carbon atoms, red: oxygen atoms, orange: phosphor atoms, blue: nitrogen atoms.

Molecular dynamics simulations have been carried out in order to study the molecular mechanisms underlying the interaction between the herbicidal molecules and a PLPC bilayer. Figure 5 displays snapshots at the end of 250 ns simulations with 28 molecules of (A) nonanoic acid, (B) sarmentine and (C) sorgoleone. From this figure, it can be seen that only sorgoleone, the largest molecule under investigation, is able to interact with every part of the lipid hydrophobic tails, while nonanoic acid and sarmentine are too short to significantly interact with the center of the bilayer.

**FIGURE 6:**
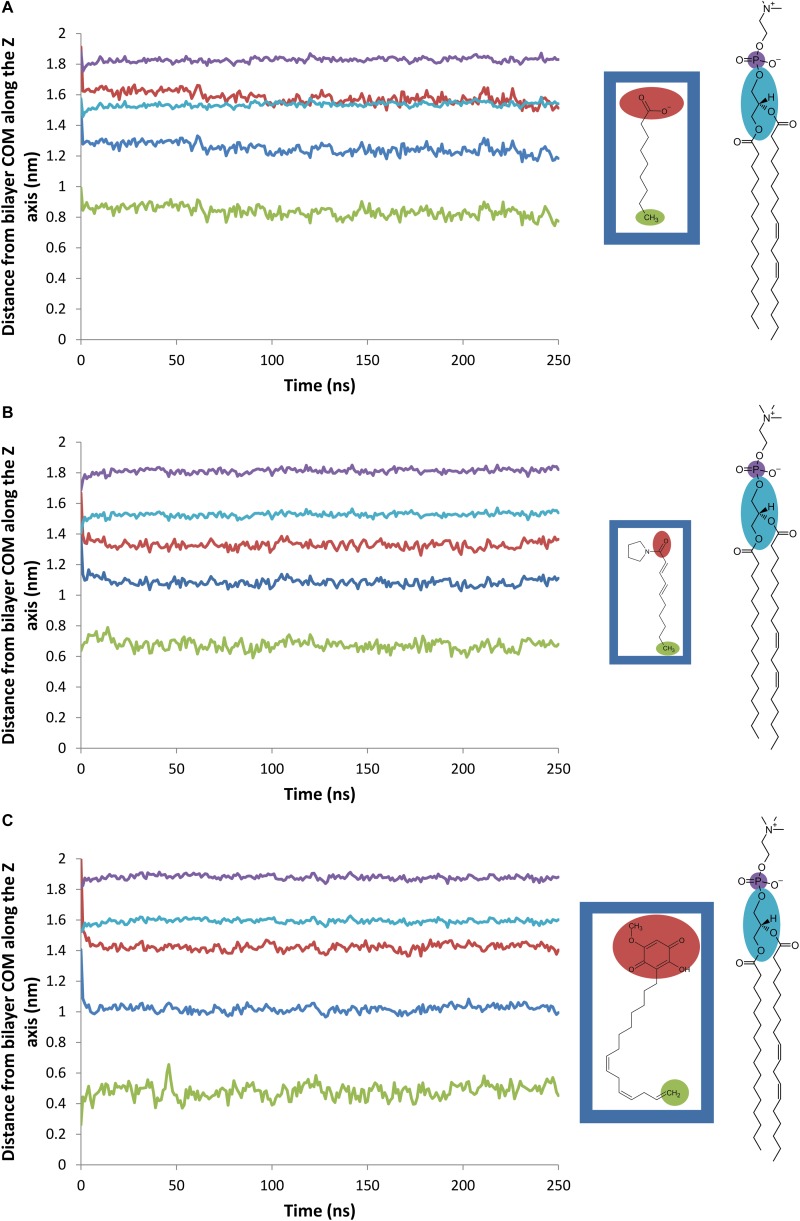
Evolution of the average distance between various atomic groups and the PLPC bilayer center of mass along the *Z*-axis (nm). Blue line: herbicide (**A**: nonanoic acid, **B**: sarmentine, and **C**: sorgoleone) COM, red line: herbicide (**A**: nonanoic acid, **B**: sarmentine, and **C**: sorgoleone) polar part (defined as shown on the inset), green line: last methyl group of herbicide (**A**: nonanoic acid, **B:** sarmentine, and **C**: sorgoleone) alkyl chain, violet line: PLPC phosphorus atoms, cyan line: PLPC glycerol groups.

The average distance between the center of mass (COM) of the bilayer and various groups of the natural herbicides along the *Z*-axis is plotted on Figure 6A–C for nonanoic acid, sarmentine and sorgoleone, respectively. The average distance between the bilayer COM and the phosphorus (P) atoms of the lipid heads is also plotted, as well as the average distance between the bilayer COM and the glycerol backbone COM. From these figures, it appears that each molecule has its own insertion pattern. The last carbon atom of the alkyl chain of sorgoleone is the most deeply buried into the hydrophobic core of the bilayer, while nonanoic acid has the shallowest one. This is not surprising, as sorgoleone has the longest alkyl chain, followed by sarmentine and then nonanoic acid. Concerning the polar moiety of each molecule, it is observed that acid group of nonanoic acid is located at the level of the glycerol backbone. Its inability to penetrate deeper into the hydrophobic core of the bilayer is likely due to its negative net charge. For sorgoleone and sarmentine, on the other hand, the polar part is located slightly deeper into the bilayer. Finally, it can be noted that the P atoms – bilayer COM distance and the glycerol backbone COM – bilayer COM distance are significantly higher for simulations with sorgoleone, which indicates that this molecule induces a thickening of the bilayer.

**FIGURE 7:**
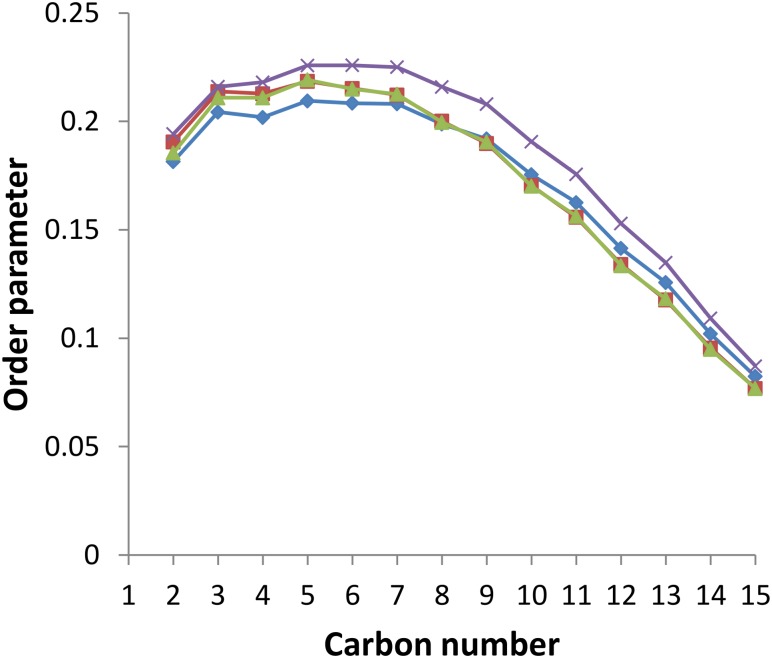
Order parameter for each carbon atom in the palmitoyl chain of PLPC in the presence or absence of herbicidal molecules. Blue line: pure PLPC, red line: PLPC + nonanoic acid (5:1 molar ratio), green line: PLPC + sarmentine (5:1 molar ratio), violet line: PLPC + sorgoleone (5:1 molar ratio).

This thickening effect of sorgoleone on the lipid bilayer is due to its ordering properties illustrated on Figure 7, which shows the order parameter of each carbon atom from the palmitoyl chain during the last 100 ns of simulation for all systems. It is observed that sorgoleone indeed induces a strong ordering effect for carbon 1 to carbon 13 of the palmitoyl chain. Interestingly, nonanoic acid and sarmentine seem able to increase, but to a lesser extent than sorgoleone, the order parameter of the first 7 carbon atoms only. Similar trends, but less marked, are also observed for the linoleoyl chain (data not shown). The lipid hydrophobic core region affected by the presence of the herbicide molecules seems thus related to the penetration depth of the last carbon atom of their alkyl chain. Deeper it is, higher the rigidification effect is and larger the lipid region where the rigidification occurs is. Surprisingly, the presence of *trans* unsaturations in sarmentine and *cis* unsaturations in sorgoleone, which could have a fluidizing effect on the packing of the lipid chains (Roach et al., 2004), seems to not have an impact on the bilayer parameter order.

## Discussion

In this study, the interactions occurring between natural herbicides and model membranes made of PC lipids were investigated. Phospholipids are one of the major components of plant plasma membranes (PPM), and PC is their most common phospholipid [(Murphy et al., 2011) and references cited therein]. It is also reported that palmitic and linoleic acids are the main fatty acids found in PPM phospholipids [(Murphy et al., 2011) and references cited therein]. Hence, PLPC was used in ITC experiments as well as for MD simulations. However, for fluorescence studies, DPPC was used in order to explore the impact of the molecules under investigation on lipid bilayers in both gel and liquid phases as well as their effect on phase transition temperature. It would not have been possible to study PLPC liposomes at their Tm and below with fluorescence measurements, as PLPC has a reported Tm of about −18.5°C.(Morrow et al., 1996) DPPC Tm is around 41.5°C, (Lewis and McElhaney, 1992) which is in good agreement with the laurdan GP curve of pure DPPC liposomes from this work (Figure 3).

From the fluorescence assays, we demonstrated that nonanoic acid and sarmentine have little influence on the lipid order, both at the level of the bilayer hydrophilic/hydrophobic interface (Laurdan experiments) and at the level of the hydrophobic core (DPH experiments), and on the phase transition temperature at the concentration tested here. In a recent work, C8 and C10 fatty acids only had a minor effect on DPPC vesicles phase transition temperature at a lipid:fatty acid molar ratio of 1:1 (Arouri et al., 2016). This is well in line with the absence of significant effect observed for nonanoic acid (lipid:fatty acid ratio of 5:1) in this study. Sarmentine was only able to induce a very slight decrease in membrane hydrophobic core fluidity, at the highest concentration (100 μM). Only sorgoleone had dramatic effects on model membranes properties. Its ability to increase DPPC Tm and order, as well as the strong decrease of DPPC hydrophobic core fluidity indicate that sorgoleone has an overall rigidifying effect on lipids. Alkylresorcinols with a structure similar to sorgoleone were recently found to have such impact on lipid bilayers, (Zawilska and Cieślik-Boczula, 2017) in agreement with the data presented here.

We clearly demonstrated that the investigated compounds do interact with PLPC LUVs using ITC. Their respective affinity for such bilayers is ranked as follows: sorgoleone > sarmentine > nonanoic acid. This is meaningful when interpreting fluorescence results: the weaker partitioning of nonanoic acid and sarmentine into liposomes partly explains why these molecules had almost no impact on the studied fluorescence parameters.

With our MD approach, natural herbicides are embedded in the lipid bilayer from the beginning of the simulations. MD data confirmed that sorgoleone induces an ordering and a thickening effect on the PLPC bilayer. Interestingly, it also showed that nonanoic acid and sarmentine slightly increase the order parameter of the upper part of lipids. This effect could stem from an alkyl chain length mismatch between the herbicidal molecules and PLPC. In addition, MD gave interesting details about these interactions at the molecular level. The deeper location of sorgoleone within the bilayer could partially explain its higher impact on lipid properties when compared to nonanoic acid, for instance. Moreover, sorgoleone polar head comprising an alcohol, two carbonyl and one methoxy, could have a higher propensity to form intermolecular electrostatic interactions and H-bonds with distinct lipid headgroups/glycerol residues. By this way, sorgoleone molecules can act as an anchor condensing the lipids at the level of their polar heads that gives rise to a rigidification of the hydrocarbon region as well, as suggested for the rigidifying effect of sphingomyelin (Shinitsky and Barenholz, 1978). The presence of *cis* unsaturations at the end of the sorgoleone chain seems to not be able to break the anchoring effect of the polar head.

From these considerations, it appears that nonanoic acid and sarmentine have lower potency to affect lipid bilayer properties than sorgoleone. However, it should be reminded that model membranes used in this study were made of PC lipids only. This lipid species is a major component of plant plasma membranes, but specific interactions of nonanoic acid or sarmentine with other lipid species cannot be ruled out. Nonetheless, nonanoic acid and sarmentine spontaneously bind to PLPC liposomes in ITC assays. Hence, it is possible that the potential target on which they exert their toxicity is embedded in membranes. If both molecules have a common target located in membrane, the higher affinity of sarmentine for lipids might at least partly explain its higher toxicity compared to nonanoic acid (Dayan et al., 2015). However, lipid affinity should not be the only factor for nonanoic acid toxicity. Indeed, the phytotoxicity of fatty acids is maximal when they are made of 9–11 carbon atoms (Fukuda et al., 2004) while the affinity of fatty acids for lipid vesicles increases when increasing the number of carbon atoms (Pernille et al., 2001), suggesting that toxicity and lipid affinity are not fully related.

From there, there are two options: (1) fatty acids phytotoxicity is not related to interactions with lipid bilayers or (2) an unknown feature of their mode of action is lowered when alkyl chain length is increased. This putative feature might be an alkyl chain length mismatch between the fatty acids and the membrane lipids. It has been reported that the degree of mismatch between alkyl chain length of lysophospholipids and membrane lipid chain length is proportional to the bilayer perturbation caused by lysophospholipids (Henriksen et al., 2010). Hence, long chain fatty acids may have a high affinity for lipid bilayers but a low chain length mismatch with them, while short chain fatty acids have a high degree of mismatch with lipid bilayers but a low affinity for them. According to this assumption, middle chain length fatty acids would benefit from intermediate levels of affinity and mismatch, with C9 to C11 presenting optimal combinations of these two factors and thus exhibiting the highest ability for lipid bilayer perturbation and eventually the highest phytotoxicity. Effects of such alkyl chain length mismatches were suggested by our MD simulations but could probably not be efficiently probed with our fluorescent assays. Techniques that enable lipid order measurements with a resolution of a single methyl group such as ^2^H NMR could be used to detect such ordering-disordering effect due to alkyl chain length mismatch.

The ordering effect that sorgoleone exerts on lipid bilayers is expected to have some consequences on membrane functions. It has been suggested that small molecules such as phytochemicals (Ingólfsson et al., 2014) or anesthetics (Cantor, 1997, 2003; Heimburg and Jackson, 2007) could modulate the activity of membrane-embedded proteins through global perturbations of the lipid environment. Interestingly, Hejl and Koster have shown that sorgoleone inhibits the H^+^-ATPase activity (Hejl and Koster, 2004). This transmembrane enzyme is responsible for the electrochemical gradient that enables metabolites uptake by the plant cells. A decreased activity of this crucial protein, potentially due to changes in lipid environment properties, would thus have a major impact on cell viability. The relationship between H^+^-ATPase activity and plant lipid environment has been recently reviewed (Morales-Cedillo et al., 2015). It is reported that modifications in membrane lipid composition are related to alterations in enzymatic activity. For instance, the presence of several sterols, molecules known for their ordering effect, (Schuler et al., 1991; Bernsdorff and Winter, 2003) induces an inhibition of H^+^-ATPase. Albeit no straightforward correlation between membrane fluidity and H^+^-ATPase activity has been established, this tends to indicate that a link could exist between the ability to alter lipid bilayer properties and the modulation of proton-pumping activity could exist. Further research is, however, needed in order to understand how the lipid environment and its modification by molecules such as sorgoleone could impact this enzyme.

## Author Contributions

MD and M-LF are the project leaders and contributed equally to the work. SL performed the experiments and the overall data analysis and wrote the manuscript. FD and LL provided technical advice. All authors revised the manuscript.

## Conflict of Interest Statement

The authors declare that the research was conducted in the absence of any commercial or financial relationships that could be construed as a potential conflict of interest.

## Abbreviations

DPPC: 1,2-Dipalmitoyl-sn-glycero-3-phosphocholine
ITC: isothermal titration calorimetry
LUV: large unilamellar vesicle
MD: molecular dynamics
MLV: multilamellar vesicle
PLPC: 1-palmitoyl-2-linoleoyl-sn-glycero-3-phosphocholine.
