# Supplementary material for: Interactions Between Natural Herbicides and Lipid Bilayers Mimicking the Plant Plasma Membrane
Source: Front Plant Sci. 2019 Mar 18;10:329. doi: 10.3389/fpls.2019.00329 (PMC6431664; doi:10.3389/fpls.2019.00329)
Supplement: Supplementary file 1 [file Data_Sheet_1.PDF]

Supplementary Information

Interactions between natural herbicides and lipid bilayers mimicking the plant plasma membrane

S. Lebecque, L. Lins, F. E. Dayan, ML. Fauconnier, M. Deleu

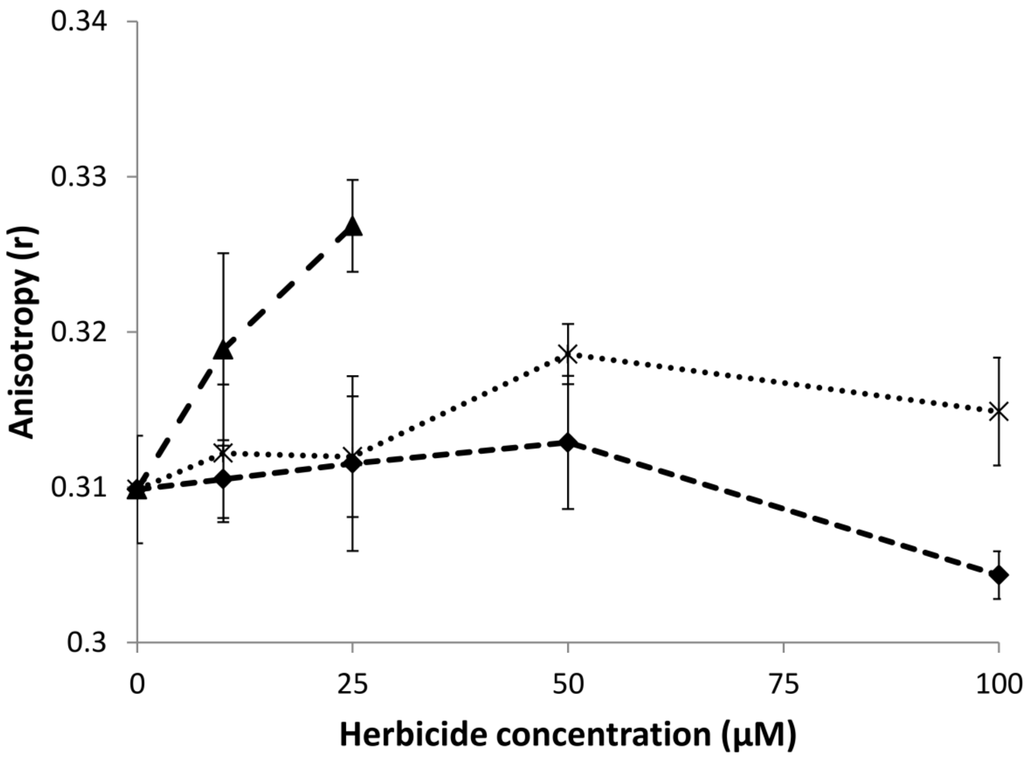

Figure S1. Evolution of DPH anisotropy for DPPC LUV (50 µM) mixed with increasing concentrations of natural herbicides at 35°C (◆: DPPC + nonanoic acid, ×: DPPC + sarmentine, ▲: DPPC + sorgoleone).
